# Uncovering the impact of COVID-19 on the place of death of cancer patients in South America

**DOI:** 10.1590/0102-311XEN057423

**Published:** 2023-12-04

**Authors:** Doris Durán, Renzo Calderon Anyosa, Belinda Nicolau, Jay S. Kaufman

**Affiliations:** 1 Faculty of Medicine and Health Sciences, McGill University, Montreal, Canada.; 2 Faculty of Dentistry, McGill University, Montreal, Canada.

**Keywords:** Neoplasms, COVID-19, Cause of Death, Neoplasias, COVID-19, Causas de Morte, Neoplasias, COVID-19, Causas de Muerte

## Abstract

The COVID-19 pandemic has significantly impacted healthcare systems worldwide, especially on the management of chronic diseases such as cancer. This study explores the effects of COVID-19 on cancer mortality trends in Brazil, Chile, and Peru. The monthly age-standardized mortality rates in different places of death (hospital/clinic or home) were estimated using vital statistics and death certificate databases. An interrupted time series analysis was performed for each country, using the date of lockdown implementation as the intervention point. Overall cancer mortality rates reduced after the implementation of pandemic restrictions, with a significant decrease in Brazil. In total, 75.3%, 55.4%, and 45.7% of deaths in Brazil, Peru, and Chile, respectively, occurred in hospitals. After lockdowns were implemented, at-home deaths increased in all countries, and in-hospital deaths correspondingly decreased only in Chile. Our results suggest that COVID-19 has significantly affected rates of cancer mortality and place of death in Latin America.

## Resumo

A pandemia de COVID-19 impactou significativamente os sistemas de saúde ao redor do mundo, especialmente no manejo de doenças crônicas, como o câncer. Este estudo explora os efeitos da COVID-19 nas tendências de mortalidade por câncer no Brasil, Chile e Peru. As taxas de mortalidade mensais padronizadas por idade em diferentes locais de morte (hospital/clínica ou domicílio) foram estimadas usando estatísticas vitais e bancos de dados de atestados de óbito. Uma análise de série temporal interrompida foi realizada para cada país, tendo como ponto de intervenção a data de implementação do *lockdown*. As taxas gerais de mortalidade por câncer reduziram após a implementação das restrições, com uma queda significativa no Brasil. No total, 75,3%, 55,4% e 45,7% dos óbitos no Brasil, Peru e Chile, respectivamente, ocorreram em hospitais. Depois da implementação dos *lockdowns*, as mortes em domicílio aumentaram em todos os países, e as mortes hospitalares diminuíram de forma correspondente apenas no Chile. Nossos resultados sugerem que a COVID-19 afetou significativamente as taxas de mortalidade por câncer e o local de morte na América Latina.

## Resumen

La pandemia de COVID-19 impactó significativamente los sistemas de salud de todo el mundo, sobre todo en el manejo de enfermedades crónicas, como el cáncer. Este estudio explora los efectos de la COVID-19 en las tendencias de mortalidad por cáncer en Brasil, Chile y Perú. Las tasas de mortalidad mensuales estandarizadas por edad en diferentes locales de fallecimiento (hospital/clínica o domicilio) se estimaron utilizando estadísticas vitales y base de datos de certificados de defunción. Se realizó un análisis de serie temporal interrumpida para cada país, teniendo como punto de intervención la fecha de implementación del *lockdown*. Las tasas generales de mortalidad por cáncer redujeron tras la implementación de las restricciones, con una disminución significativa en Brasil. En total, el 75,3% de los óbitos ocurrieron en hospitales en Brasil, el 55,4% en Perú y el 45,7% en Chile. Tras la implementación del *lockdown*, las muertes domiciliarias aumentaron en todos los países, y las muertes hospitalarias solo redujeron de forma correspondiente en Chile. Nuestros resultados sugieren que la COVID-19 afectó significativamente las tasas de mortalidad por cáncer y el local del fallecimiento en América Latina.

## Introduction

The COVID-19 pandemic, caused by SARS-CoV-2, has affected health systems worldwide, prompting questions about the impact of mobility restriction measures and hospital overcrowding on chronic disease management, including cancer. After noticing the swift spread of different variants and the high mortality rates in countries affected early by the virus ^1^ ^,^ ^2^, many countries quickly took action to prevent similar scenarios from happening in their own territories. Governments worldwide enforced mobility restrictions, implemented in significantly different ways depending on the country ^3^.

Latin America was one of the most aﬀected regions in the world with highly heterogeneous COVID-19 responses and fatality counts ^4^. But, in addition to COVID-19 mortality, the impact of the pandemic can be seen in other disease indicators ^5^ ^,^ ^6^. For example, in cases where COVID-19 has been described as a more signiﬁcant risk factor for death, such as immunity-debilitating diseases or cancer treatment and therapy.

The question about the intersection of COVID-19 and cancer has been raised several times during the pandemic. Even though extensive research has already been produced ^7^ ^,^ ^8^ ^,^ ^9^ ^,^ ^10^ ^,^ ^11^ ^,^ ^12^, evidence for this topic is still scarce in Latin America. Studies such as the one by Vásquez Rosas et al. ^13^ reported a decrease in cancer screening and delays in cancer treatments in several Latin American countries. This is further supported by Cuadrado et al. ^7^, who also reported this situation in Chile. Additionally, in a Colombian cohort of oncologic patients, a higher mortality risk was observed among cancer patients with an active disease ^14^.

The pandemic also influenced the general population’s place of death. Changes in healthcare-seeking behaviors, due to fear or to the unavailability of services may have affected where people died. Edwards & Wohl ^15^ compared the probability of dying at home before (2014-2019) and after the pandemic (2020-2021) in North Carolina (United States). They pointed out a 23% increase in all-cause deaths at home and also observed that this risk varied depending on race/ethnicity: by the end of 2020, at-home deaths had increased by 65% for Hispanics.

The extent to which the place of death for people with cancer varied among Latin American countries and how these deaths were affected by the COVID-19 pandemic is unknown. Data collection efforts in this region have yielded widely available and good quality vital statistics, especially data on deaths, which creates an opportunity to explore the effects of the COVID-19 pandemic on different aspects of death in several countries. In this study, we aimed to evaluate cancer mortality trends during the COVID-19 pandemic in Brazil, Chile, and Peru, comparing at-home and in-hospital deaths.

## Methods

We identified the number of deaths from July 2019 to July 2021 using vital statistics and death certificate databases from Brazil (Mortality Information System - SIM, acronym in Portuguese), Chile (Department of Statistics and Health Information - DEIS, acronym in Spanish), and Peru (National Information System of Deaths - SINADEF, acronym in Spanish). Our analysis included people with malignant neoplasms as underlying causes of death. We calculated the monthly age-standardized mortality rates (ASMR) in each country and reported places of death (hospital/clinic or home). Since these were no major differences between sexes, we chose to report overall mortality in our study. However, the Supplementary Material 1 (https://cadernos.ensp.fiocruz.br/static//arquivo/suppl-1-e00057423_5787.pdf) shows the analyses by sex.

We conducted an interrupted time series for each country using the date of implementation of lockdowns or when strict mobility measures took place (March 2020 for the three countries) as the intervention point. We then fitted a linear model, considering a variable for the time of the interventions, a dummy variable for pre- and post-intervention periods, and an interaction term between them. To assess the changes according to the place of death, we included a variable indicating whether deaths happened at home or in hospitals and included interaction terms of this variable with the time of the interventions and with the indicator of the pre- and post-intervention periods. Confidence intervals were obtained parametrically with the maximum likelihood estimation of the standard error of the coefficients from the regression.

As sensitivity analyses, we first tested a lag effect, changing our intervention date from March 2020 to April 2020 to explore if the effect is only observed at the intervention or if it is delayed in time by an induction period. Then, we used a lead control, changing the intervention date to December 2019, to verify that the effect started at the intervention and not before.

## Results

During the observed period, 3,753,804 deaths with reported place of death occurred. Of these, 13.1% and 21.8% deaths were due to cancer and COVID-19, respectively. Table 1 shows that, from 2020 to 2021, 30,128 (1%) and 2,425 (0.5%) people in Brazil and Peru, respectively, had both cancer and COVID-19 on their death certificates. This information was not available for Chile. In all countries, most recorded deaths were of men and adults over 40 years of age. The highest proportion of cancer deaths was observed in Chile (20.2%). Additionally, 32.8% of Peru’s deaths in 2020-2021 were caused by COVID-19, while in Brazil and Chile 20.2% and 20.6% of deaths were caused by the virus, respectively. In total, 75.3%, 55.4%, and 45.7% of deaths in Brazil, Peru, and Chile, respectively, happened in hospitals. In all countries, men had a higher proportion of deaths (55.7%, 53.6%, and 58.3% in Brazil, Peru, and Chile, respectively). In Peru, 61.1% of people who died in hospitals were men, whereas the percentages were 53% and 55.9% in Brazil and Chile, respectively. Death certificates that reported both COVID-19 and cancer as causes of death were most frequent for in-hospital deaths, both in Brazil (1.3% were reported as in-hospital deaths and 0.1% as at-home deaths) and Peru (1% referred to in-hospital deaths and 0.1% to at-home).

**Table 1:** Overall characteristics of death records from Brazil, Chile, and Peru according to the place of death. July 2019 to July 2021.

Characteristics	Brazil [n (%)]				Chile [n (%)]				Peru [n (%)]			
	Home	Hospital	Other	Overall	Home	Hospital	Other	Overall	Home	Hospital	Other	Overall
	(n = 579,752)	(n = 2,292,114)	(n = 171,252)	(n = 3,043,118)	(n = 111,751)	(n = 107,385)	(n = 16,556)	(n = 235,692)	(n = 190,779)	(n = 262,977)	(n = 21,238)	(n = 474,994)
Sex												
Female	254,724 (43.9)	1,055,966 (46.1)	33,539 (19.6)	1,344,229 (44.2)	57,335 (51.3)	47,357 (44.1)	4,587 (27.7)	109,279 (46.4)	89,643 (47.0)	102,358 (38.9)	6,193 (29.2)	198,194 (41.7)
Male	324,908 (56.0)	123,4042 (53.8)	137,214 (80.1)	1,696,164 (55.7)	54,416 (48.7)	60,003 (55.9)	11,968 (72.3)	126,387 (53.6)	101,086 (53.0)	160,597 (61.1)	15,042 (70.8)	276,725 (58.3)
Missing	120 (0.0)	2,106 (0.1)	499 (0.3)	2,725 (0.1)	0 (0.0)	25 (0.0)	1 (0.0)	26 (0.0)	50 (0.0)	22 (0.0)	3 (0.0)	75 (0.0)
Age group (years)												
≤ 19	8,109 (1.4)	78,952 (3.4)	15,271 (8.9)	102,332 (3.4)	380 (0.3)	2,502 (2.3)	746 (4.5)	3,628 (1.5)	4,359 (2.3)	10,992 (4.2)	2,156 (10.2)	17,507 (3.7)
20-39	31,720 (5.5)	135,855 (5.9)	63,651 (37.2)	231,226 (7.6)	1,949 (1.7)	3,695 (3.4)	3,804 (23.0)	9,448 (4.0)	7,635 (4.0)	15,573 (5.9)	5,283 (24.9)	28,491 (6.0)
40-59	95,103 (16.4)	465,263 (20.3)	42,649 (24.9)	603,015 (19.8)	10,723 (9.6)	17,242 (16.1)	4,589 (27.7)	32,554 (13.8)	24,560 (12.9)	61,679 (23.5)	5,193 (24.5)	91,432 (19.2)
60-79	212,557 (36.7)	964,006 (42.1)	29,233 (17.1)	1,205,796 (39.6)	39,983 (35.8)	49,871 (46.4)	4,597 (27.8)	94,451 (40.1)	68,079 (35.7)	118,540 (45.1)	5,397 (25.4)	192,016 (40.4)
≥ 80	230,164 (39.7)	598,666 (26.1)	17,222 (10.1)	846,052 (27.8)	58,716 (52.5)	34,075 (31.7)	2,819 (17.0)	95,610 (40.6)	85,978 (45.1)	56,105 (21.3)	3,191 (15.0)	145,274 (30.6)
Missing	2,099 (0.4)	49,372 (2.2)	3,226 (1.9)	54,697 (1.8)	0 (0.0)	0 (0.0)	1 (0.0)	1 (0.0)	168 (0.1)	88 (0.0)	18 (0.1)	274 (0.1)
Year												
2020	325,143 (56.1)	1,157,136 (50.5)	97,993 (57.2)	1,580,272 (51.9)	59,568 (53.3)	56,087 (52.2)	10,186 (61.5)	125,841 (53.4)	94,710 (49.6)	122,098 (46.4)	9,815 (46.2)	226,623 (47.7)
2021	254,609 (43.9)	1,134,978 (49.5)	73,259 (42.8)	1,462,846 (48.1)	52,183 (46.7)	51,298 (47.8)	6,370 (38.5)	109,851 (46.6)	96,069 (50.4)	140,879 (53.6.)	11,423 (53.8)	248,371 (52.3)
Underlying cause of death												
COVID-19	14,459 (2.5)	595,839 (26.0)	4,343 (2.5)	614,641 (20.2)	9,946 (8.9)	37,433 (34.9)	1,075 (6.5)	48,454 (20.6)	18,663 (9.8)	133,515 (50.8)	3,409 (16.1)	155,587 (32.8)
Neoplasm	75,310 (13.0)	317,881 (13.9)	3,946 (2.3)	397,137 (13.1)	34,353 (30.7)	12,499 (11.6)	748 (4.5)	47,600 (20.2)	30,609 (16.0)	17,550 (6.7)	681 (3.2)	48,840 (10.3)
Other	489,983 (84.5)	1,378,394 (60.1)	162,963 (95.2)	2,031,340 (66.8)	67,452 (60.4)	57,453 (53.5)	14,733 (89.0)	139,638 (59.2)	141,507 (74.2)	111,912 (42.6.)	17,148 (80.7)	270,567 (57.0)
Multiple causes of death *: cancer and COVID-19												
No	578,963 (99.9)	2,262,923 (98.7)	171,104 (99.9)	3,012,990 (99.0)					190,542 (99.9)	260,816 (99.2)	21,211 (99.9)	472,569 (99.5)
Yes	789 (0.1)	29,191 (1.3)	148 (0.1)	30,128 (1.0)					237 (0.1)	2,161 (0.8)	27 (0.1)	2,425 (0.5)

* Multiple causes of death could only be assessed in records from Brazil and Peru. These two countries publish all causes of death present in death certificates. Here, we show the frequency of the mention of cancer (any type) and COVID-19, regardless of the location reported in the certificates, i.e., causes leading to death in part I or causes contributing to death in part II.

**Figure 1:**
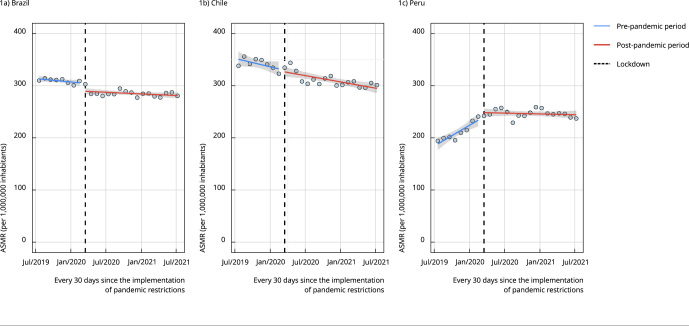
Overall cancer mortality rate of people over 50 years of age. Brazil, Chile, and Peru, 2019-2020.

Figure 1 shows the interrupted time series for the overall cancer mortality. After pandemic restrictions were implemented, the overall cancer ASMR was immediately reduced, with -14.9 (95% confidence interval - 95%CI: -25.2; -4.8) deaths and -5.3 (95%CI: -23.4; 12.8) deaths per million people in Brazil and Chile, respectively. In both countries, especially Chile, trends before the pandemic pointed toward a reduction in cancer mortality. In Peru, however, the trend for cancer mortality was increasing during the pre-intervention period, with 9.3 (95%CI: -5.5; 24.1) more deaths per million people - and this may have occurred due to strategies that were implemented to improve death registration (see *Discussion*). Therefore, our immediate change estimate must be interpreted cautiously, as it is probably misestimating the real change.

ASMR: age-standardized mortality rate. Note: the fitted lines correspond to the least squares linear regression.

**Figure 2:**
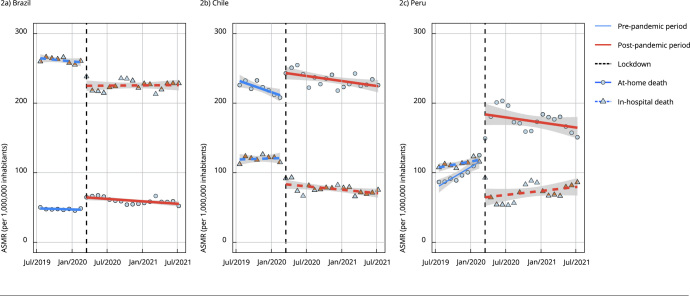
Interrupted time series for cancer mortality in different places of death *. Brazil, Chile and Peru, 2019-2021.

Figure 2 shows the interrupted time series for cancer mortality by place of death. We observed three different pre-intervention scenarios: in Brazil, in-hospital cancer mortality was higher, while in Chile, at-home cancer deaths prevailed. Before the pandemic, Peru’s death registrations seem to have been distributed evenly between hospitals and homes, but as aforementioned, the high slope could reflect changes in the completeness of death registrations.

ASMR: age-standardized mortality rate. Note: the fitted lines correspond to the least squares linear regression; intervention: implementation of mobility restrictions. * Place of death: home or hospital, as registered in death certificates

Figure 2 also shows that after the implementation of mobility restrictions to prevent COVID-19, including lockdowns, a similar pattern occurred in all countries: at-home cancer deaths increased, and cancer deaths in hospitals decreased. Brazil, Chile, and Peru had 17.9 (95%CI: 11.5; 24.3), 34.7 (95%CI: 19.2; 50.2), and 63.3 (95%CI: 37.8; 88.9) more at-home deaths per million people, respectively. Conversely, hospital mortality rates decreased by -33.1 (95%CI: -46.0; -20.2), -38.0 (95%CI: -49.5; -26.4), and -55.3 (95%CI: -75.7; -34.9) deaths per million people.

In Brazil, there were -50.9 (95%CI: -64.9; -37.0) less in-hospital cancer deaths per million people than at-home cancer deaths. This difference was -72.7 (95%CI: -91.4; -53.9) and -118.6 (95%CI: -150.0; -86.8) less deaths per million people in hospitals compared to homes, for Chile and Peru respectively.

The sensitivity analyses we conducted supported our findings. When the date of the intervention was moved forward, the effect we found was sustained, as would be expected in a dynamic situation such as a pandemic. Moving the intervention date backwards did not change mortality rates by place of death. For Peru, however, the lead control analyses showed an increase in mortality even before the pandemic, supporting our cautious approach when interpreting immediate changes in overall mortality. These findings, available and briefly discussed in the Supplementary Material 2 (https://cadernos.ensp.fiocruz.br/static//arquivo/suppl-2-e00057423_1368.pdf), concur with the policies in force in Peru to improve death registration.

## Discussion

Our results highlight the drastic changes that occurred in 2020 and 2021 due to the COVID-19 pandemic, impacting death rates, particularly cancer deaths, in Brazil, Peru and Chile. In the three countries, a distinct shift in the reported place of cancer deaths emerged during this period, being characterized by an increase in deaths at home and a simultaneous decrease in deaths in hospitals. Chile presented the most balanced shift, where the rise in at-home fatalities was almost equal to the decrease of in-hospital deaths.

This paper raises important questions about the impact of the pandemic on cancer mortality and on the healthcare systems of Latin American countries. Further research is needed to investigate the reasons for the shift in the reported at-home and in-hospital deaths, and to better understand the effects of the pandemic on cancer mortality. It is also important to unravel the potential implications of this shift regarding access to health care and health outcomes for different subpopulations.

We propose two main possible explanations for the observed changes in cancer mortality during the COVID-19 pandemic. First, public health measures such as mobility restrictions changed access and care-seeking behaviors, delaying hospital presentation and changing preferences about the place of death for cancer patients. Secondly, COVID-19 deaths may have presented as a competing event for cancer death, which could be the speciﬁc explanation for the unbalanced shift of cancer deaths observed in Brazil.

COVID-19 as a competing event, in turn, can be due to COVID-19 causing death per se in cancer patients and reducing cancer mortality (*factual* decrease). Alternatively, because even in cases where the person died of cancer complications, if they had a positive COVID-19 test, and some of the surveillance guidelines would identify them as COVID-19 deaths, they would not count as cancer deaths in oﬃcial statistics (*registration* decrease). The extent of the latter situation is unknown and will vary by country orders and pandemic control regulations in place.

Our research was limited by data availability. Since the death registration database does not contain clinical information, we could not ascertain the exact cancer stage patients were experiencing when they died and whether they were undergoing treatments. Furthermore, we could not assess individual preferences regarding place of death, which may have influenced the different mortality rates at home and in hospitals. For example, López-Valcárcel et al. ^16^ reported that European patients with cancer preferred to die at home. We do not know if this is also the case in Latin America. Notably, our data showed higher at-home cancer mortality in Chile before the pandemic, which aligns with the country’s policy of universal palliative care coverage for cancer patients, implemented in 2005 ^17^ ^,^ ^18^.

Our study may also have measurement errors, particularly in our evaluation of data from Peru, due to the country’s historically low death registration coverage ^19^. However, due to Peru’s recent significant improvement in registration processes ^20^, our results may have overestimated the changes in mortality rates after the implementation of mobility restrictions in the country, possibly due to the underestimation of mortality rates before these measures. This was supported by the lead control, which showed an increasing mortality when we moved the intervention date to December 2019. The efforts to improve death registration in Peru, through the use of an electronic reporting web platform and many other tools, was recently challenged by failures in the security features, which allowed for data entry of people who had not died ^21^. The SINADEF is currently under review, and its future is uncertain ^22^ ^,^ ^23^.

This study strengths include the use of high-quality whole population data from Chile and Brazil, and moderate-quality data from Peru ^24^. The information about place of death presented in death certificates is complete and accurate, providing high-quality data for our analyses ^24^ ^,^ ^25^. Furthermore, the coverage of death registrations in Brazil and Chile is close to 100% ^26^. Also, over 95% of completeness of cause of death in both countries ^27^ contribute to the robustness of our findings. Although the effects of COVID-19 on various systems may have delayed death registration processes, leading to the underestimation of mortality at the onset of the pandemic, we did not anticipate the backlog to change our results, as this problem was likely resolved in subsequent periods.

Our analysis considered the underlying cause of death per official statistics. We found that less than 1% of death reports from Brazil and Peru mentioned both COVID-19 and cancer as causes of fatality. These deaths, mostly registered as in hospitals, support our competing event hypothesis. However, it is uncertain if these are *factual* or due to *registration* discrepancies. Since the “any mention” approach could cause researchers to overcount deaths, thereby altering the total number of deaths ^28^, we plan to conduct further research using alternate methods to explore this intersection.

Furthermore, our study underscores the crucial role of accurate information on causes of death, which is not only important in the current crisis, but also in the management of future emergencies, as it aids the effective tracking, surveillance, and long-term impact monitoring of different fatality causes.

Our study is the first to address this question in Latin America, allowing for a unique comparison of three countries with different health care systems and approaches to the COVID-19 pandemic, quantifying a foreseeable consequence of the pandemic, and assessing an unaddressed part of the intersection between COVID-19 and cancer. We hope this work helps public health practitioners, end-of-life care professionals and palliative care experts prepare for future emergencies. Understanding these findings can help adapt and allocate resources to meet the evolving needs of health care even beyond the pandemic.
